# Deubiquitinating Enzyme USP12 Regulates the Pro-Apoptosis Protein Bax

**DOI:** 10.3390/ijms232113107

**Published:** 2022-10-28

**Authors:** Hae-Seul Choi, Eun-Su Lim, Kwang-Hyun Baek

**Affiliations:** Department of Biomedical Science, CHA University, Seongnam 13488, Gyeonggi-do, Korea

**Keywords:** deubiquitination, ubiquitin-proteasome system, yeast two-hybrid screening, protein half-life

## Abstract

The Bax protein is a pro-apoptotic protein belonging to the Bcl-2 family, involved in inducing apoptosis at the mitochondrial level. Regulating the protein levels of Bax is essential to enhancing apoptosis. In the current study, we ascertained the presence of deubiquitinating enzymes (DUBs) associated with Bax by performing the yeast two-hybrid screening (Y2H). We determined that ubiquitin-specific protease 12 (USP12), one of the DUBs, is associated with Bax. The binding of USP12 to Bax shows the interaction as a DUB, which regulates ubiquitination on Bax. Taken together, we believe that USP12 regulates Bax by detaching ubiquitin on K63-linked chains, indicating that USP12 affects the cellular functions of Bax, but it is not related with proteasomal degradation. The half-life of the Bax protein was determined by performing the site-directed mutagenesis of putative ubiquitination sites on Bax (K128R, K189R, and K190R). Of these, Bax (K128R and K190R) showed less ubiquitination; therefore, we compared the half-life of Bax (WT) and Bax K mutant forms in vitro. Interestingly, Bax (K189R) showed a higher ubiquitination level and shorter half-life than Bax (WT), and the (K128R and K190R) mutant form has a longer half-life than Bax (WT).

## 1. Introduction

Accumulated malfunctioning proteins lead to adverse outcomes in the affected organism. Thus, protein regulation pathways play an important role in organisms. The ubiquitin-mediated protein degradation pathway is one such typical pathway of numerous protein regulation pathways existing in the organism [1]. More than two such ubiquitin attachments result in mono- or polyubiquitin chains on the target protein [2,3], leading to protein degradation or imparting other functions that affect DNA damage response, translation, stress response, and so on [3]. The ubiquitination pathway progresses via a series of enzyme reactions [4] comprising the consecutive reactions of E1, E2, and E3 enzymes. E1 is an activating enzyme involved in the ATP-dependent response forming a thiol ester bond that connects the glycine of ubiquitin to the cysteine residue of the E1 enzyme. E2 is a conjugating enzyme, which transfers ubiquitin to E3. Finally, E3 acts as a ligase that transfers and links ubiquitin to the lysine residue of the target protein by forming an amide bond [5]. This pathway generates an isopeptide bond in the middle of the ubiquitin and lysine residue of the substrate [6]. Only occasionally, ubiquitin binds to serine, threonine, and cysteine on the substrate. The ubiquitin, combined with its substrate, forms a polyubiquitin chain [7,8]. Ubiquitination is initiated on one of the seven lysine residues or methionine 1 site, which constitutes different information. Ubiquitin-mediated modifications have different ubiquitination chains. For example, the K6-linked ubiquitin chain regulates DNA repair, K11-linked ubiquitin chain is related to human cell cycle control and proteasomal degradation, K27-linked ubiquitin chain regulates DNA damage response, K29-linked ubiquitin chain mediates Ub-fusion degradation, K33-linked ubiquitin chain regulates T cell receptor (TCR) signaling, K48-linked ubiquitin chain leads to degradation by the 26S proteasome, and K63-linked ubiquitin modifies target proteins in various reactions, such as DNA damage response, stress response, lysosomal targeting, etc. [9,10]. Ubiquitination is performed through the E1, E2, and E3 cascade, whereas deubiquitinating enzymes have a function which detaches the ubiquitin or defense polyubiquitination through a cascade reaction of the E1, E2, and E3 enzymes [11]. Similarly, a reversal pathway of ubiquitination regulates the level of target proteins. The deubiquitinating enzyme (DUB) prevents target proteins from degradation by the 26S proteasome and modifies the ubiquitination system by detaching the ubiquitin from the lysine sites of target proteins. The DUB has several subfamilies. However, since each subfamily has a different effect on target proteins, the complete DUB mechanisms are not fully understood [12]. Many DUBs act as a hydrolase that cut off the amide bond between ubiquitin and the target protein [13]. The ubiquitination and deubiquitination processes need to maintain a balance in organisms. Approximately 100 DUBs are encrypted in the human genome and can be classified into at least nine families: ubiquitin-specific proteases (UPSs), ovarian tumor proteases (OTUs), ubiquitin C-terminal hydrolases (UCHs), Machado-Josephin domains (MJDs), MIU-containing novel DUB family (MINDY), JAB1/MPN/MOV34 (JAMMs), monocyte chemotactic protein-induced proteases (MCPIPs) and ZUFSP/ZUP1 [14], and the papain fold peptidase of dsRNA viruses and eukaryotes (PPPDE) [15]. Apoptosis is the programmed cell death which depends on both extrinsic and intrinsic signaling pathways. The extrinsic pathway is initiated by a death receptor on the cell surface, whereas the intrinsic pathway is initiated by stress signals, such as UV damage, hypoxia, oxidative stress, and DNA damage [16]. Stress signals induce the apoptosis pathway, which is initiated by a pro-apoptotic protein at the mitochondrial level. Bax, which is located in the cytosol in a non-apoptosis state, initiates translocation on the mitochondrial outer membrane after oligomerization with Bak [17,18], a result of which is an increase in the mitochondrial membrane outer membrane permeability (MOMP). The protein cytochrome c is located in the inner membrane then moves into the cytosol [19]. Cytochrome c plays an important role in activation of pro-caspase-9, followed by the activation of caspases-3, -6, and -7 and the induction of apoptosis [20,21]. Bax is an initiator of the intrinsic apoptosis pathway. Therefore, determining how to control Bax would provide an approach to control apoptosis. In 2018, a study found that the silencing of USP12 in prostate cancer regulates the TP53 signaling pathway and affects the upregulation of Bax [22]. However, it is still not confirmed whether USP12 regulates Bax via its DUB activity, and the protein–protein interaction between two proteins have not been discovered. In a study carried out in 2010, it was confirmed that Bax is ubiquitinated, and the ubiquitination level and protein stability of Bax can be regulated by IBTDC2, one of E3 ligases, which increases Bax ubiquitination [23]. It is known that the Parkin E3 ligase has its enzymatic activity on Bax [24]. Additionally, Parkin prevents stress-induced translocation of Bax to the mitochondria [24]. Evaluating the results of the yeast two-hybrid (Y2H) screening revealed that ubiquitin-specific protease 12 (USP12) is associated with Bax, and the GST pull-down and binding assays revealed the interaction between Bax and USP12. Therefore, at present, we will further investigate ubiquitination and deubiquitination of Bax and the cellular interplay between USP12 and Bax.

## 2. Results

**Figure 1:**
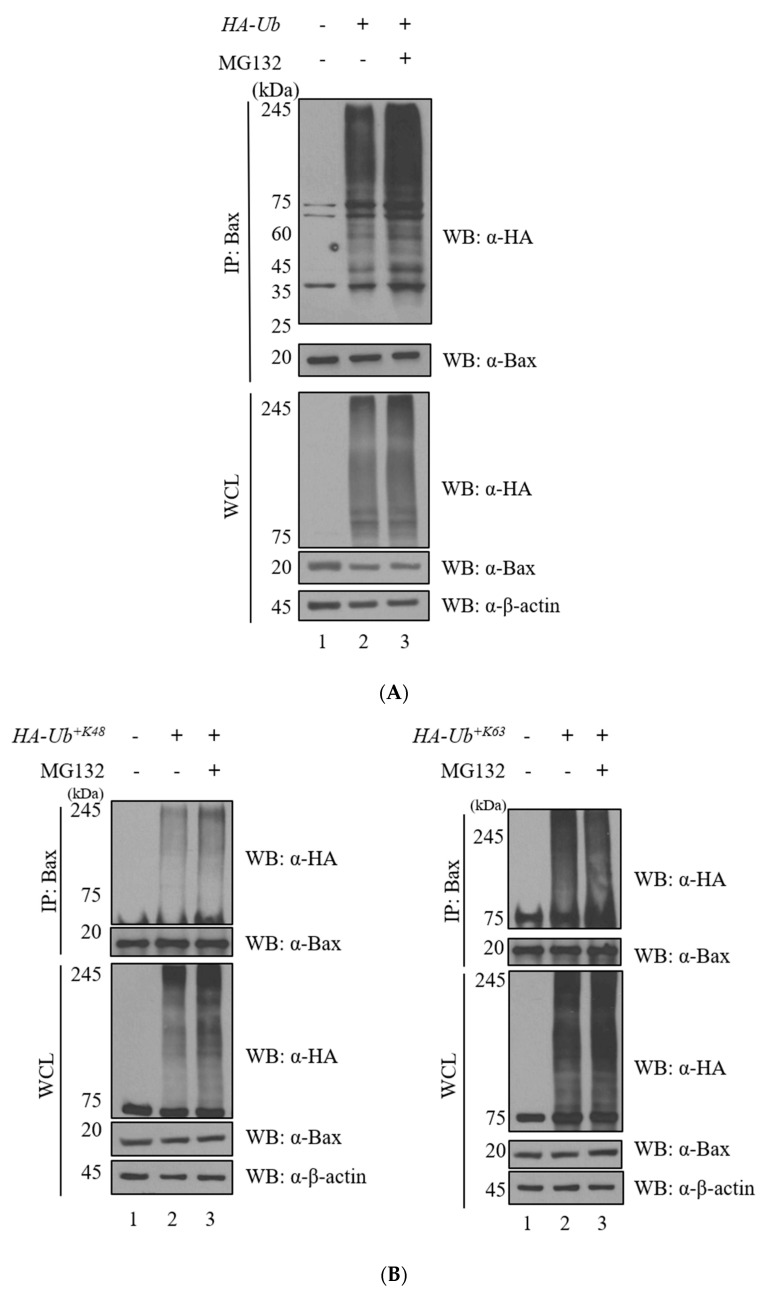
Bax is polyubiquitinated. (**A**) To confirm the regulation of Bax by UPS, the ubiquitination assay was performed using Myc-Bax and HA-ubiquitin. (**B**) The same analysis was performed using the lysine mutations of *Myc-Bax (WT*) and *HA-Ub^+K48^* and *HA-Ub^+K63^* to determine the ubiquitin chain position in Bax ubiquitination and to investigate whether ubiquitination of Bax is related to proteasomal degradation, DNA damage response, stress response, and other pathways, such as lysosomal targeting.

**Figure 2:**
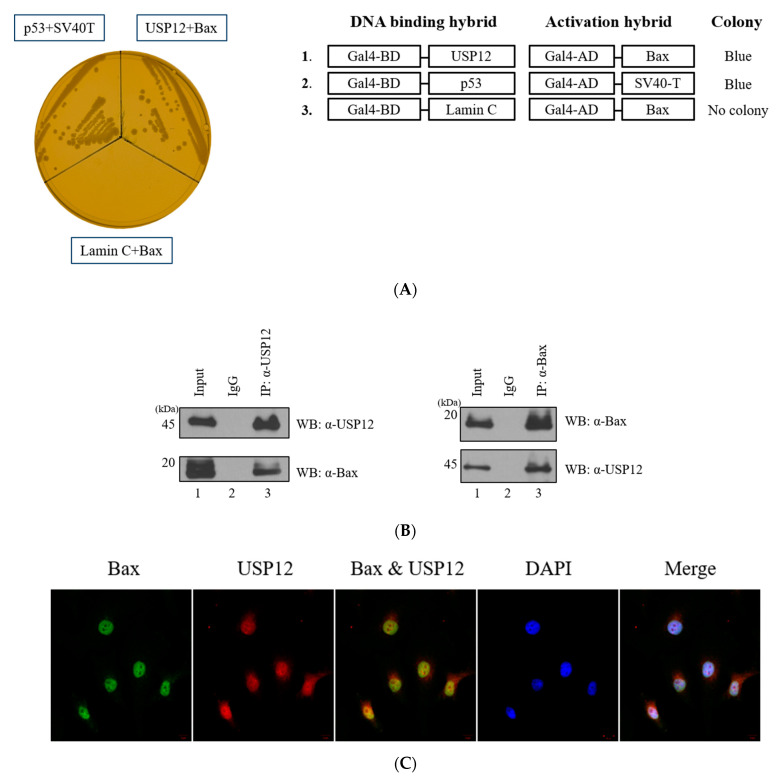

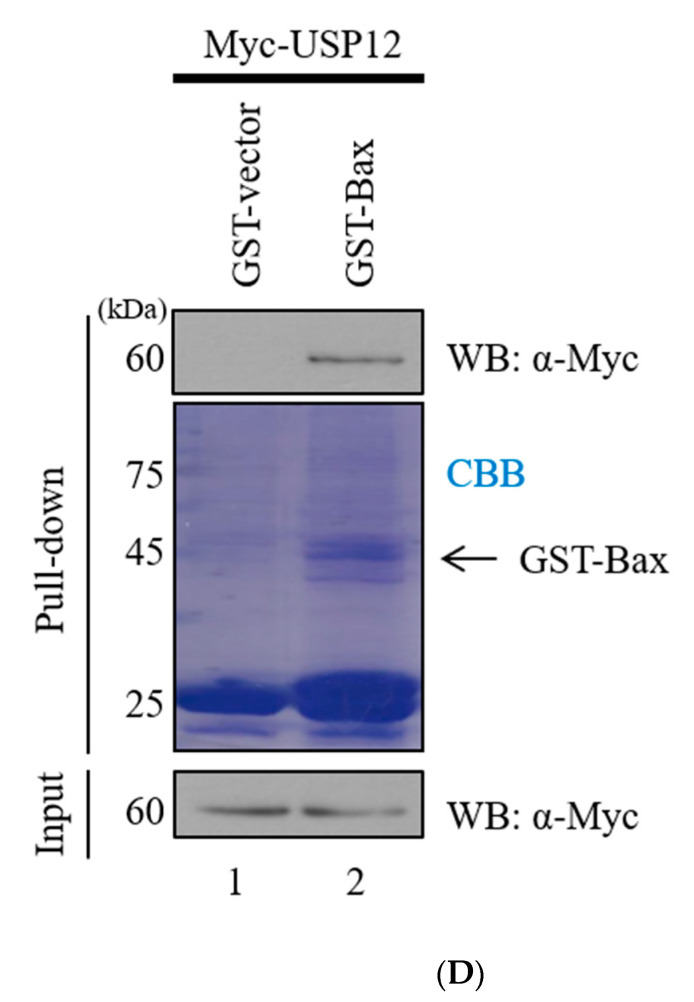
Binding assay between USP12 and Bax. (**A**) Results of a previous study, where we determined that USP12 interacts with Bax. p53 + SV40: a positive control (**B**) GST pull-down assay was performed for checking interactions between USP12 and Bax. (**C**) Immunocytochemical analysis was performed to identify the co-localization of Bax and USP12. (**D**) GST pull-down assay was performed with pGEX-4T-3-Bax.

**Figure 3:**
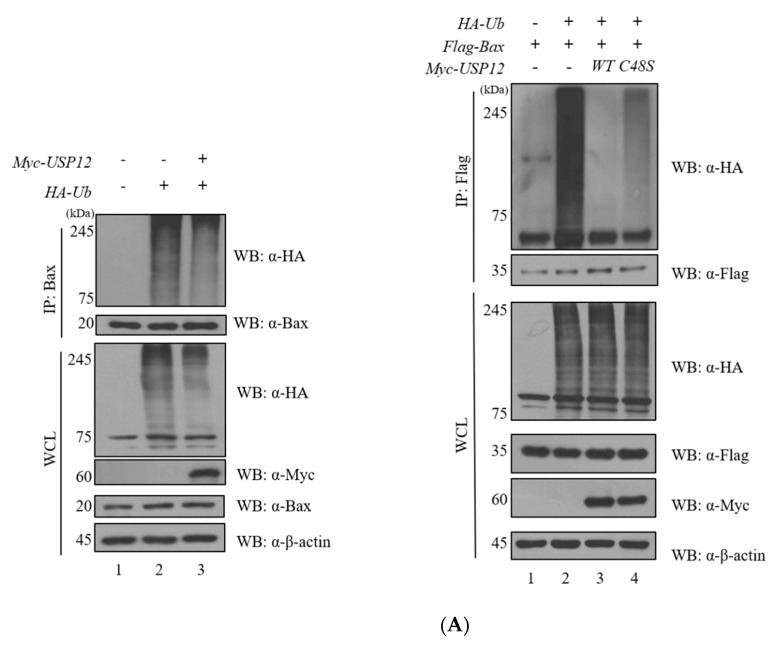

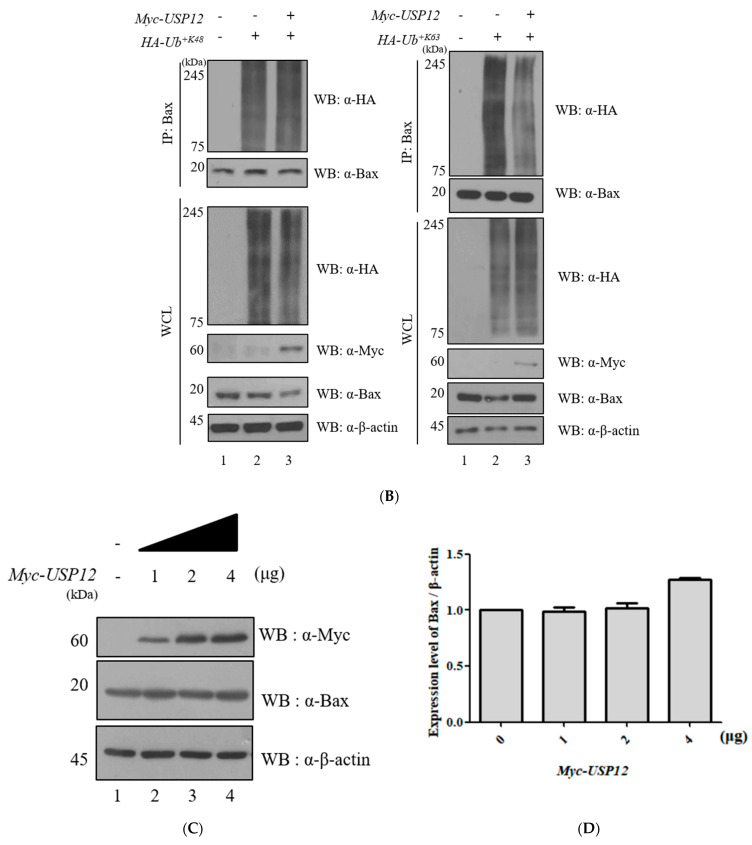

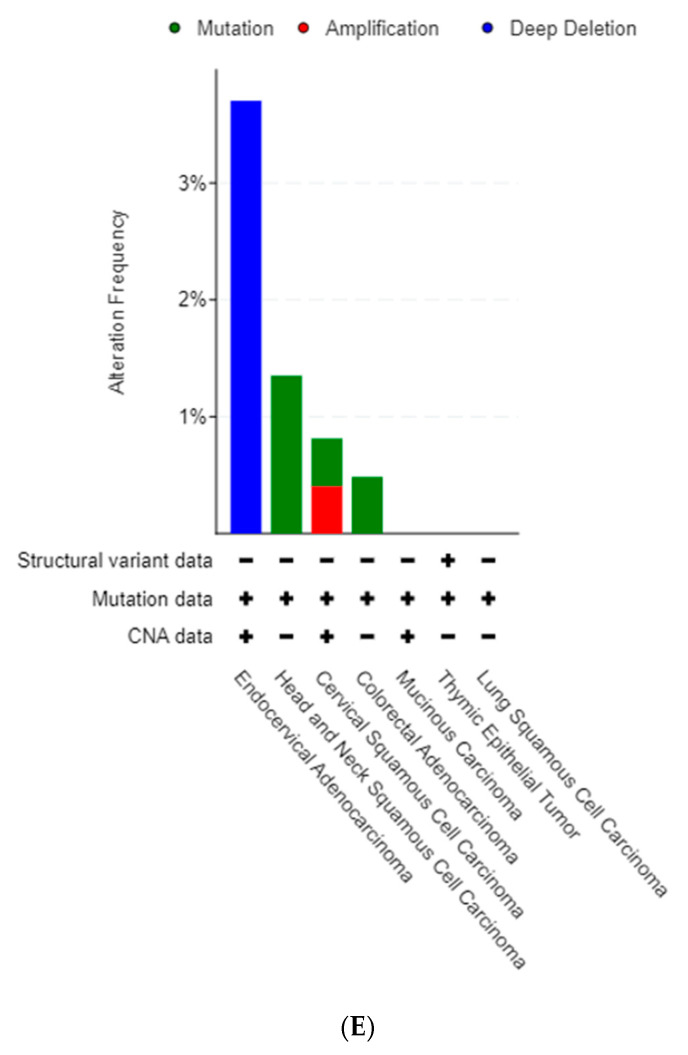
Deubiquitination assay of Bax by USP12. (**A**) The DUB assay was performed to check enzymatic activity on the ubiquitination chain of Bax, using *Flag-USP12, Myc-USP12*, and *Myc-USP12 (C48S)*. (**B**) The same assay was repeated using *HA-Ub^+K48^* and *HA-Ub^+K63^*, to determine whether the lysine-linked ubiquitin chain formed in Bax is regulated by DUB. (**C**) To determine whether the protein expression level of Bax is regulated by USP12, Western blotting of Flag-USP12 and Bax was performed in a dose-dependent manner of Flag-USP12. (**D**) Experiments were performed at least three times for the quantitation of Western blot results (n = 3). (**E**) Summary of alteration frequency for USP12 via cBioPortal.org. Graph reports % of samples harboring the indicated alteration per study. Green: mutation; Red: Amplification; Blue: Deletion.

**Figure 4:**
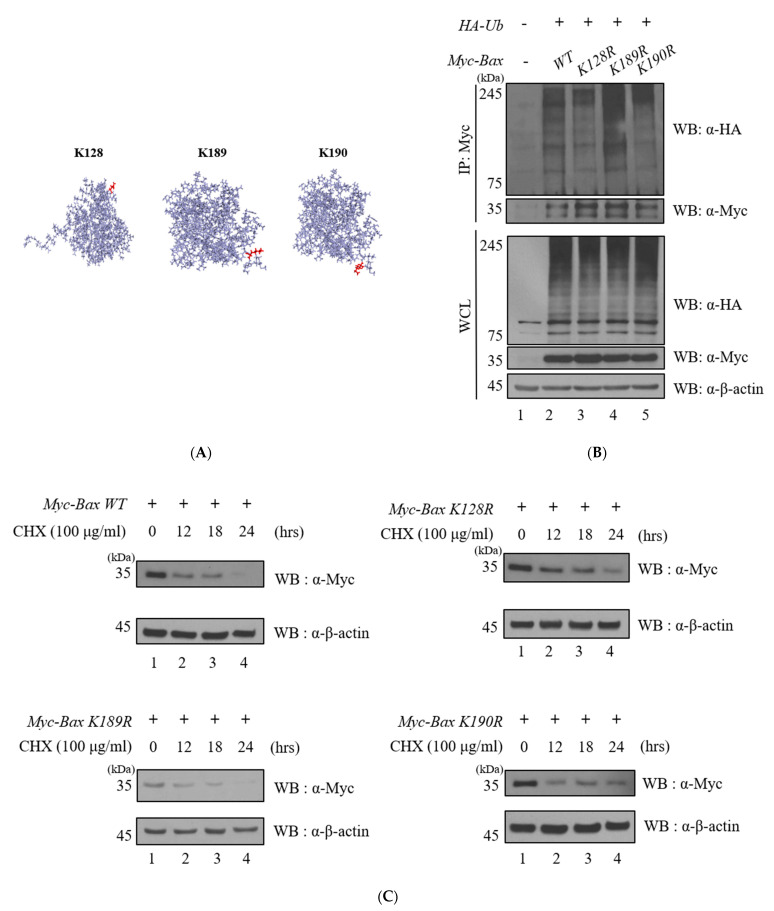

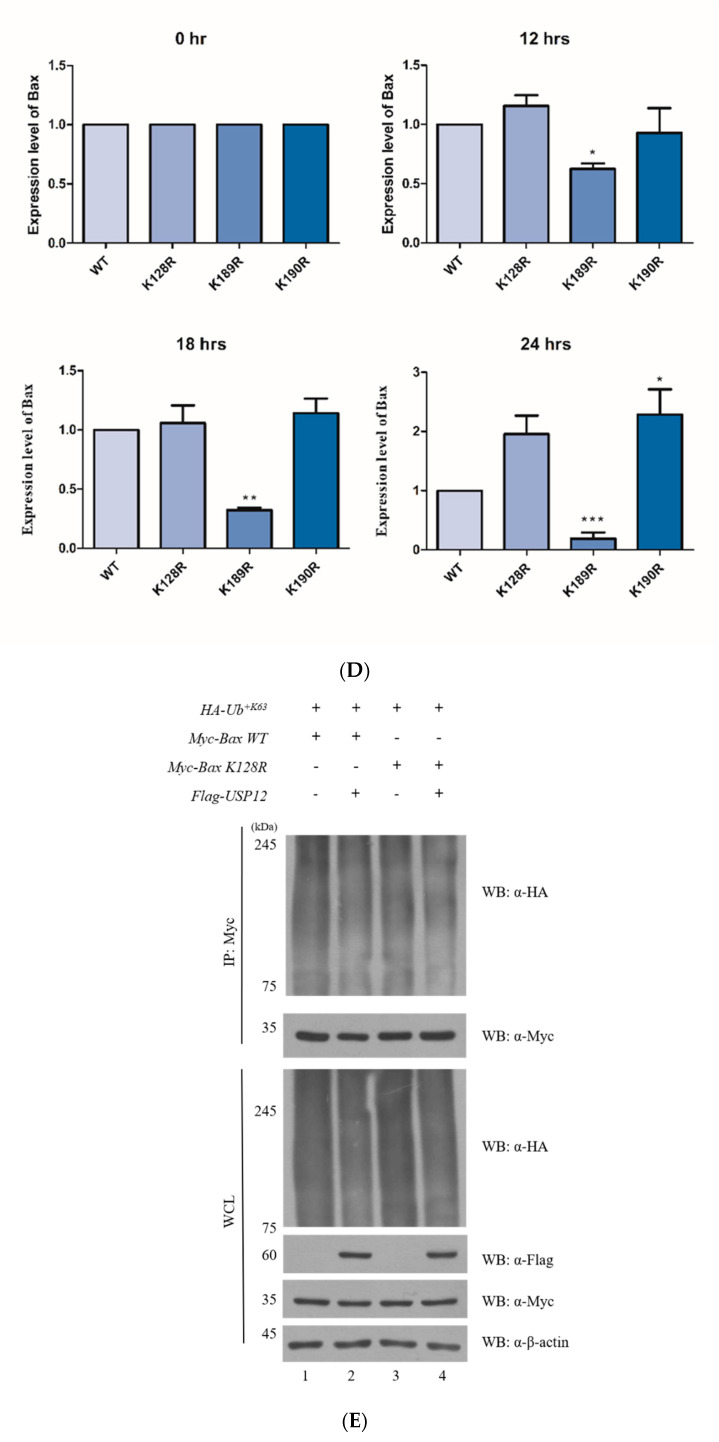
Lys mutant forms of Bax. (**A**) Location of Lys implemented through the “PyMOL” program. (**B**) The ubiquitination assay was performed using single K mutant of Myc-Bax WT (K128R, K189R, and K190R). (**C**) HeLa cells were treated with CHX (0, 2, 4, and 8 hrs). Protein levels were determined by Western blotting. (**D**) Experiments were performed at least three times for quantitation of Western blot results (n = 3, * *p* < 0.05, ** *p* < 0.01, *** *p* < 0.001). (**E**) Deubiquitination assay of Myc-Bax WT and K128R was performed using HA-Ub^+K63^.

Ubiquitination assays were performed to investigate the ubiquitination of Bax (Figure 1, Supplementary Figure S1). Y2H screening was performed to identify the interaction between two proteins (Figure 2). Immunoprecipitation, immunocytochemistry, and GST pull-down assays revealed that the two proteins bind to each other (Figure 2). The deubiquitination assay for Bax with USP12 further revealed that the deubiquitinating enzyme USP12 has DUB activity on Bax but does not regulate the protein stability of Bax (Figure 3). However, it regulates the K63-linked polyubiquitin chain, which is known to be associated with DNA damage and stress response on Bax. Therefore, we performed the ubiquitination assay of Bax and Bax-K mutant forms (K128R, K189R, and K190R) to determine the ubiquitination site of Bax (Figure 4).

### 2.1. Bax Is Regulated by the USP System

The ubiquitination assay was performed to identify whether Bax undergoes ubiquitin-mediated degradation (Figure 1A). Our results indicate that the Bax protein is regulated by ubiquitination. Depending on which polyubiquitin chain is formed, the regulation of target protein by ubiquitination is related to proteasomal degradation (K48) or other mechanisms. The same assay was performed using HA-ubiquitin lysine mutant forms (HA-Ub^+K48^ and HA-Ub^+K63^) to identify the location of the ubiquitinating lysine (Figure 1B).

### 2.2. USP12 Is a Binding Partner of Bax

We performed Y2H screening to identify the DUB of Bax. We determined that Bax binds to USP12 (Figure 2A,B). Immunocytochemical analysis was also carried out to determine the localization of Bax and USP12 in HeLa cells (Figure 2C). In addition, the GST pull-down assay was performed to check for the interaction between Bax and USP12 (Figure 2D). Our results indicate that USP12 and Bax are mostly located in the nucleus, with a minimal amount found in the cytoplasm. These two proteins interact via both direct and indirect methods and are located in the same regions in HeLa cells.

### 2.3. USP12 Exerts DUB Activity on Bax

The assessment for the DUB activity of USP12 on Bax was achieved by the deubiquitination assay for Bax, and the catalytically inactive form (C48S) of USP12 showed decreased DUB activity on Bax (Figure 3A). USP12 does not regulate the stability of Bax, and we therefore investigated whether USP12 regulates the cellular functions of Bax by deubiquitination. Our study examined whether polyubiquitin chains are attached on Bax and USP12 regulates ubiquitin chains on Bax. To investigate the polyubiquitin chains on Bax, the ubiquitination assay was performed using lysine mutant constructs of *HA-ubiquitin* (*HA-Ub^+K48^* and *HA-Ub^+K63^*). Our results revealed that Bax has polyubiquitin chains on K48 and K63 (Figure 3B), and USP12 exerts DUB activity on the K63-linked polyubiquitin chain (Figure 3B) but not on the K48-linked polyubiquitin chain (Figure 3B). K48-linked polyubiquitin chain is known to regulate proteasome-mediated protein degradation, whereas K63-polyubiquitination is associated with the DNA damage response, stress response, and lysosomal targeting [9,10]. Western blotting performed using varying concentration of Myc-USP12 to evaluate the dose-dependent expression levels of Bax revealed no increase in the protein levels of Bax when exposed to dose-dependent concentrations of Myc-USP12 (Figure 3C,D). Thus, we deduce that although Bax is regulated by the UPS system, the protein expression levels of Bax are not regulated by the binding partner USP12.

It was confirmed through the TCGA database that the frequency of mutations and deep deletions of *USP12* are high in cervical cancers (Figure 3E). Therefore, we conducted experiments on the interaction of USP12 with Bax in the cervical cancer cell line, HeLa.

### 2.4. K128 and K190 Are Polyubiquitination Sites of Bax

In order to find putative lysine sites with a high probability of ubiquitination on Bax, lysines were short-listed by referring to the BDM PUB (http://bdmpub.biocuckoo.org/ accessed on 25 June 2020), jci-bioinfo (http://www.jci-bioinfo.cn accessed on 25 June 2020), RUBI (http://old.protein.bio.unipd.it/rubi/ accessed on 25 June 2020), and Netchop (http://www.cbs.dtu.dk/services/NetChop accessed on 25 June 2020); final selection of Lys residues on the surface of Bax was achieved using the PyMOL program (https://pymol.org/2/ accessed on 25 June 2020) (Figure 4A). The ubiquitination assay was performed using the Lys mutant forms of Bax to identify the ubiquitination sites K128, K189, and K190 of Bax (Figure 4B). The site-directed mutagenesis of Myc-Bax single mutant forms (Myc-Bax K128R, K189R, and K190R) were further completed. The cycloheximide (CHX) assay was performed to compare the half-life between the wild-type Bax and the mutants K128R, K189R, and K190R. Our results confirmed that the K189R mutant has a longer half-life as compared to the wild-type (Figure 4C). Graphs were subjected to statistical analysis for calculating the protein levels (Figure 4D). As a result, it was found that K128 is a strong candidate for the ubiquitination site of Bax (Figure 4D). We performed a ubiquitination assay to confirm whether USP12 exhibits DUB activity even on the K128R mutant. The assay demonstrated that USP12 has DUB activity on Bax-K128R mutant. Interestingly, USP12 regulates the K63-linked polyubiquitin chain in the Bax-K128R mutant even though a lower ubiquitination level of Bax-K128R was shown compared to the Bax-WT (Figure 4E).

## 3. Discussion

Apoptosis, the mechanism of inducing a damaged or unrecoverable cell to annihilation, was first defined in 1972 [25]. Apoptosis is divided into two stages: the beginning stage (starting) and execution stage. The starting phase is a complex process, comprising numerous different proteins. It is initiated by “stress” inside and outside the cell [26]. Proteins of the Bcl-2 family, encompassing 25 known proteins, control the internal pathways. Within the cell, proteins belonging to this family function to stimulate a balance between pro-apoptosis and anti-apoptosis [27,28]. The Bcl-2 family proteins are divided into three subfamilies, according to the number of BH (Bcl-2 Homology) domains. The first subfamily includes the anti-apoptotic proteins Bcl-2, Bcl-xL, Bcl-w, MCL-1, and BFL-1. These proteins possess four BH domains (BH1-4). The other two groups are pro-apoptotic proteins possessing three BH domains (BH1-3), represented by Bax, Bak, Bok, and BH3-only proteins, respectively; as implied by their name, proteins of the latter group are characterized by the presence of only the BH3 domain [29]. The tumor suppressor p53 exerts its anti-neoplastic activity primarily through the induction of apoptosis. The cytosolic localization of endogenous wild-type or trans-activation-deficient p53 is necessary and sufficient for apoptosis. p53 directly activates the pro-apoptotic Bcl-2 protein Bax in the absence of other proteins, leading to the permeabilization of the mitochondria and the initiation of the apoptotic program. p53 also releases both pro-apoptotic multidomain proteins and BH3-only proteins that are sequestered by BCL-xL. The transcription-independent activation of Bax by p53 occurs with similar kinetics and concentrations to levels produced by the activated Bid. When p53 is accumulated in the cytosol, it functions as analogous to the BH3-only subset of pro-apoptotic Bcl-2 proteins to activate Bax and trigger apoptosis [30]. In our experiments, USP12 regulated the pro-apoptotic protein, Bax, as a DUB. We confirmed the interaction between USP12 and Bax via binding and GST pull-down assays. The transfection of *Flag-USP12* was achieved in a dose-dependent manner to identify USP12 as a stabilizer for Bax. However, our results indicate that USP12 does not control the protein expression levels of Bax via deubiquitination. To verify our prediction, we focused on the K63-linked polyubiquitin chains on Bax. For the DUB assay, mutant ubiquitins were applied to investigate whether USP12 regulates the K63-linked polyubiquitin chains of Bax. We observed that the DUB activity of USP12 results in decreased ubiquitination levels on HA-Ub^+K48^ and HA-Ub^+K63^, thereby confirming that USP12 does not regulate the K48-linked polyubiquitin chain, (which is known to be involved in proteasomal degradation) but regulates the K63-linked polyubiquitin chains of Bax. We further performed the site-directed mutagenesis of Bax to specify lysine sites that are ubiquitinated and to extend the half-life of Bax. The lysine mutant forms (K128R, K189R, and K190R) were used for the ubiquitination assay, and we demonstrated that K128R is an influential ubiquitination site of Bax. From previous data, it is known that USP12 regulates the DNA damage response, cell signaling, and protein localization [31]. According to developmental and adult tissue physiology, it affects the cellular functions of Bax, which is not associated with 26S proteasomal degradation. Bax-dependent apoptosis was induced with interaction of apoptosis on the BH domains and translocation into mitochondrial outer membrane with TM [32,33]. If each domain was blocked by another protein (E1B 19K), the apoptosis pathway induced by stress signals was interrupted [34]. USP12 is reportedly associated with DNA damage repair [35,36]. A previous report states that USP12 and UAF form a complex with an enzymatic effect, after which the enzymatic effect of USP12 and UAF complex on target protein is stimulated by WDR20 [37,38]. Previous reports showed that the overexpression of USP12 does not significantly affect cell proliferation, cell cycle, and other functions of the USP12 knockdown state [39,40]. Notch signaling is important in developmental and adult tissue physiology and is known to regulate cell death to some degree. USP12 silencing interferes with the Notch activity. USP12 activates UAF1 at the biochemical level, causing the deubiquitination of Notch in cells and in vitro. These results indicate that stored regulation of the Notch signaling pathway promotes or inhibits cell differentiation, proliferation, or death [41]. One study reported that USP12 modifies mHTT-mediated neurodegeneration and toxicity in neurons obtained from rodents and patients with Huntington’s disease [42]. USP12 also regulates pAkt in prostate cancer cell lines and stabilizes two Akt phosphatases (PHLPP and PHLPPL) [43]. The ubiquitination of the pro-apoptotic protein Bax is regulated by USP12, but USP12 does not affect the protein stabilization of Bax. Consequently, USP12 regulates the K63-linked polyubiquitin chains of Bax. The effects of K63-linked polyubiquitination and signaling processes need to be further studied. In addition, deep deletions and mutations of *USP12* with high frequency were observed in cervical cancer cells through informatics analysis. Future studies of the anticancer activity of Bax and USP12 proteins are recommended in cervical cancer cell lines. The mutant forms of Bax have been reported in previous studies. A novel structural positional point mutation of Bax (K21) involved in the activation of Bax has already been reported [44], and cysteine 62 in Bax is known to be important for conformational activation and pro-apoptotic activity in response to H_2_O_2_-induced apoptosis [45]. In addition, a previous study reported that K128E of Bax has the potential to alter the release of cytochrome c, and the point mutations of Bax prevent apoptosis-inducing functions [46]. The importance of the role of DUBs has been emphasized, and it is known that DUB functions in apoptosis and aging-related diseases, including neurodegenerative diseases [47,48]. Research on these DUBs should therefore continue for cancers or neurodegenerative diseases. Our results indicate that USP12 shows that partial deubiquitinating activity regulates the K63-linked polyubiquitin chain of Bax. Future studies should be focused on elucidating what action is mediated by the USP12-regulation K63-linked chain of Bax (Figure 3). Lastly, a mutation in the K128 and K190 residues in Bax reduced the ubiquitination of Bax itself, and it was confirmed that this mutation regulates the half-life of Bax (Figure 4), thus defining the increased stability of the Bax protein as a potential target for promoting cell death in cancer cells and therapeutic development.

## 4. Materials and Methods

### 4.1. Construction of Expression Vectors

The full-length cDNAs for *USP12* and Bax were PCR-amplified from HeLa cells and subcloned into pcDNA3.1-6Myc vector and pCS4-Flag B vector. *USP12* (C48S) was generated from the *Myc-USP12* construct described in a previous study [37]. We used *HA-Ub*, *HA-Ub^+K48^*, and *HA-Ub^+K63^* constructs that were published in our previous study [49].

### 4.2. Cell Culture Condition, Constructs, and Transfection

HeLa cells were incubated in Dulbecco’s modified Eagle’s medium (DMEM, Gibco, Grand Island, NY, USA) supplemented with 10% fetal bovine serum (FBS, Gibco-BRL, Rockville, MD, USA) and 1% penicillin and streptomycin (Gibco-BRL, Rockville, MD, USA). The cells were grown in 5% CO₂ incubator at 37 °C. For transfection, 10 mM polyethyleneimine reagent (PEI, Polysciences, Inc., Warrington, PA, USA) (6–9 μL/μg of DNA) and 150 mM NaCl (100 μL/mL of media) were used and incubated at 37 °C for 48 h.

### 4.3. Antibodies

Anti-Flag (Sigma-Aldrich, St. Louis, MO, USA), anti-Myc (9E10 hybridoma cell media), anti-Bax (Santa Cruz Biotechnology, Santa Cruz, CA, USA), anti-USP12 (Invitrogen, Paisley, UK), anti-HA (12CA5, Roche, Basel, Switzerland), and anti-β-actin (Santa Cruz Biotechnology, Santa Cruz, CA, USA) antibodies were used for Western blotting and immunoprecipitation analyses.

### 4.4. Yeast Two-Hybrid Screening (Y2H)

AH109 yeast strain was streaked on YPD (Clontech, Palo Alto, CA, USA) agar plates and incubated at 30 °C for 3–4 days. AH109 colonies were cultured in YPD media (Clontech, Palo Alto, CA, USA) and transformed using the LiAc method. Yeast cells were transformed with USP family members as a bait and incubated in -Trp plates. After 3–4 days, colonies were transformed with a pGAD424-Bax and incubated in -Leu/-Trp minimal medium plates containing 4 mg/mL X-α-gal (Clontech, Palo Alto, CA, USA). There was a positive control for protein–protein interaction between p53 and SV-40 large T-antigen and formed blue colonies.

### 4.5. Western Blotting and Immunoprecipitation

Cells were lysed using a lysis buffer (50 mM Tris-HCl [pH 7.5], 1 mM EDTA, 10% Glycerol, 300 mM NaCl and 1% Triton X-100) and cell extracts were incubated for 20 min on ice. Then, the samples were centrifuged at 13,000 rpm at 4 °C for 20 min and supernatant was boiled with 2X SDS sample buffer for Western blotting. For immunoprecipitation assay, the cell lysates were rotated with antibodies at 4 °C overnight. Protein A/G PLUS agarose beads (Santa Cruz Biotechnology, Santa Cruz, CA, USA) were added into the cell lysates and rotated for 2 h. The samples were washed two times with wash buffer (Lysis buffer with PMSF and PIC 1:100) and resuspended at 2X SDS sample buffer.

### 4.6. GST Pull-Down Assay

GST-Bax was transformed into BL21 (DE3), the bacteria were grown until an O.D. value of 0.5 was obtained (A600), and GST and GST-Bax proteins were induced with 0.5 mM isopropyl β-d-1-thiogalactopyranoside (IPTG) (Promega, Madison, WI, USA) at 37 °C for 4 h. The protein bound to GST-Bax was washed and boiled with a 2X SDS buffer. The bound protein was detected through Western blotting, and GST and GST-Bax were visualized with a Coomassie Brilliant Blue R (SLBL7178V, Sigma-Aldrich, St. Louis, MO, USA) and G (SLBN7053V, Sigma-Aldrich, St. Louis, MO, USA) solution [50].

### 4.7. Immunocytochemistry

HeLa cells were seeded on a glass coverslip placed in a 12-well plate and incubated at 37 °C overnight, washed with phosphate-buffered saline (PBS), fixed with 4% formaldehyde for 15 min, blocked with PBS containing 5% normal goat serum for 1 hr at room temperature, and treated with primary antibodies in 1% BSA at 4 °C overnight. Cells were then washed with PBS, incubated with Alexa-Fluor-488-conjugated goat anti-mouse (1:500, Invitrogen, Carlsbad, CA, USA) or with Alexa-Fluor-568-conjugated goat anti-rabbit (1:500, Invitrogen, Carlsbad, CA, USA) for 1 hr at room temperature in the dark, washed with PBS, and stained with DAPI (1:1000). Samples were visualized under a confocal microscope (Zeiss LSM880, Carl Zeiss Microscopy GmbH, Jena, Germany).

### 4.8. Ubiquitination and Deubiquitination Assays

For ubiquitination assay, *Myc-Bax*, and *HA-Ub* were co-transfected into HeLa cells. The cell lysates were used for immunoprecipitation with an anti-Myc antibody. They were incubated with an antibody at 4 °C overnight and then for 2 hrs with protein A/G Plus-Agarose Beads (Santa Cruz Biotechnology, Santa Cruz, CA, USA). Western blotting was performed with the cell lysates. For deubiquitination assay, *Flag-USP12*, *Myc-Bax*, and *HA-Ub* were co-transfected into HeLa cells. Immunoprecipitation was performed with an anti-Myc antibody. Both assays were performed using a ubiquitination assay kit, according to the manufacturer’s manual (cat. no. UBAK-100; D&P Biotech Inc. Seoul, Korea).

### 4.9. TCGA and cBioPortal

The ’TCGA’ (https://www.cancer.gov/ accessed on 2 July 2022) and ‘cBioPortal’ (https://www.cbioportal.org/ accessed on 2 July 2022) databases were used to check the alteration frequency of *USP12* in various cancers and to select the cell line to conduct the experiment.

### 4.10. PyMOL–3D Structure

We used the “PyMOL” program (The PyMOL Molecular Graphics System, Version 1.2r3pre, Schrödinger, LLC) to project the ubiquitination sites based on the structure of Bax. The structure of the Bax protein was implemented and mutant forms were made by substituting lysines with alanines on the surface of the protein.

### 4.11. Site-Directed Mutagenesis

The mutation sites of Bax were chosen using the “PyMOL” 3D structure system. For the *Myc-Bax (K128R)* mutant, PCR was performed using *Myc-Bax (WT)* at 95 °C for 30 s, 60 °C for 30 s, and 68 °C for 5 min for a total of 12 cycles. For *Myc-Bax (K189R)* and *Myc-Bax (K190R)* mutants, PCR was performed using *Myc-Bax (WT)* at 95 °C for 30 s, 54.4 °C for 30 s, and 68 °C for 5 min, for a total of 12 cycles. The forward primers (K128R: 5′-TGC ACC AGG GTG CCG GAA-3′; K189R: 5′-ATC TGG AGG AAG ATG GGC-3′; K190R: 5′-ATC TGG AAG AGG ATG GGC-3′); and the reverse primers (K128R: 5′-TTC CGG CAC CCT GGT GCC-3′; K189R: 5′-GCC CAT CTT CCT CCA GAT-3′; and K190R: 5′-GCC CAT CCT CTT CCA GAT-3′) were used to replace the lysines with arginines.

### 4.12. Cycloheximide (CHX) Assay

HeLa and HEK293T cells were transfected with *Myc-Bax (WT)* or *Myc-Bax (K128R)* or *Myc-Bax (K189R)* or *Myc-Bax (K190R)*. After 24 hrs of incubation, HeLa and HEK293T cells were treated with CHX (1:1000, 100 mg/mL) for 0, 3, 6, and 12 hrs. Then Western blotting was performed.

### 4.13. Statistical Analysis

Statistical analysis was conducted using the *t*-test and one-way analysis of variance followed by Tukey’s multiple comparisons post hoc tests using GraphPad Prism version 5 (GraphPad Software, La Jolla, CA, USA). At least three separate tests were performed. Densitometric analysis was conducted using Image J software (Version 1.4.3, National Institutes of Health, Bethesda, MD, USA). *p* values of * *p* < 0.05, ** *p* < 0.01, *** *p* < 0.001 were deemed significant.

## Data Availability

The data presented in this study are available in the article and Supplementary Material.

## Supplementary Materials

The supporting information can be downloaded at: https://www.mdpi.com/article/10.3390/ijms232113107/s1.
