# Supplementary figures and images for: Deubiquitinating Enzyme USP12 Regulates the Pro-Apoptosis Protein Bax
Source: Int J Mol Sci. 2022 Oct 28;23(21):13107. doi: 10.3390/ijms232113107 (PMC9657785; doi:10.3390/ijms232113107)

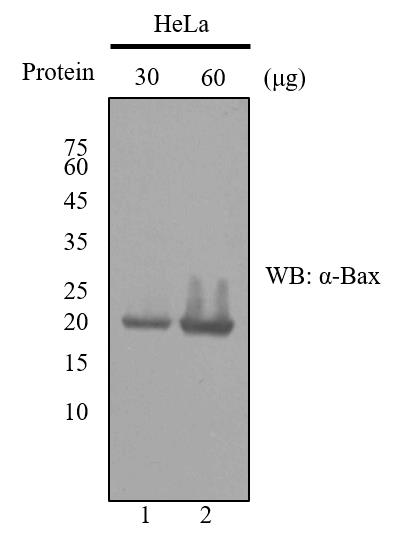

Supplement: Supplementary file 1 [file ijms-23-13107-s001.zip › ijms-1879055-supplementary Figure S1.jpg]
